# Positive religious coping acts through perception of nature and silence in its association with well-being and life satisfaction among Polish Catholics

**DOI:** 10.3389/fpubh.2022.1020007

**Published:** 2022-11-07

**Authors:** Sebastian Binyamin Skalski-Bednarz, Karol Konaszewski, Loren L. Toussaint, Arndt Büssing, Janusz Surzykiewicz

**Affiliations:** ^1^Faculty of Philosophy and Education, Catholic University of Eichstätt-Ingolstadt, Eichstätt, Germany; ^2^Faculty of Education, Cardinal Stefan Wyszyński University in Warsaw, Warsaw, Poland; ^3^Faculty of Education, University of Białystok, Białystok, Poland; ^4^Department of Psychology, Luther College, Decorah, IA, United States; ^5^Professorship Quality of Life, Spirituality and Coping, Witten/Herdecke University, Herdecke, Germany

**Keywords:** religious coping, perception of nature and silence, well-being, life satisfaction, Catholics

## Abstract

Previous studies indicate that perceptions of nature and thought-provoking silence can have positive consequences for individual functioning. The purpose of the present study was to assess the relationships between religious coping (assessed with the Brief RCOPE), perceptions of nature and silence (a subscale of the Perception of Change Questionnaire), well-being (the World Health Organization's five-item Well-Being Index) and life satisfaction (the Brief Multidimensional Life Satisfaction Scale). An online questionnaire was completed between 2021–2022 by 1,010 Polish Catholics ages 18 to 73, 61% of whom were women. Structural equation modeling showed that positive religious coping was positively related to well-being (β = 0.08, *p* = 0.011) and life satisfaction (β = 0.22, *p* < 0.001). In contrast, negative religious coping was related to reduced well-being (β = −0.07, *p* = 0.040) and life satisfaction (β = −0.25). In addition, more frequent perceptions of nature and reflective times of silence partially mediated the associations of positive religious coping with well-being (β = 0.04, *p* = 0.011) and life satisfaction (β = 0.04, *p* = 0.008). The data might suggest that interventions that help people develop an ability or awareness for nature as an exceptional encounter and may help to strengthen the ways they can utilize their religiosity as a resource and thus contribute to well-being and life satisfaction among Catholics.

## Introduction

Religion is recognized as a system that provides meaning to people's lives (1) as well as a coping strategy when it is a central source of comfort in a person's life (2). Empirical evidence has consistently shown that there are (usually weak) relationships between religion and life satisfaction and well-being in religious societies (3–8), while in rather secular societies such an association is inconsistently found, depending on the specific indicator of spirituality and the sample (9–11). Life satisfaction is a cognitive evaluation or judgment of how well–things are in terms of subjective satisfaction within specific areas of relevance (12). It is usually multidimensionally assessed. Subjective well-being, on the other hand, is an appraisal of global life situations that reflects a sense that our lives are going well, that one is happy with it. Diener, et al. (13) added to their conceptualization of well-being the individual judgement that one would not change much if one could live life again. According to the literature, religious people regularly consider themselves happier and more satisfied with their lives (13–17), although the effect sizes are usually weak and are depend on the population and the specific situation. Although most of the research has focused on Christian populations, the relationship between religion and life satisfaction and well-being appears to be consistent across cultural contexts. For example, Abdel-Khalek (18) and Tiliouine (17) have observed that religiosity predicts meaning and life satisfaction, and religiosity is associated with better health and well-being and less anxiety in Muslims. However, in the present study we focused exclusively on the Christian religion and, more specifically, on Catholics. We chose this sample because, according to previous studies, greater theologically conservative views in Christianity were associated with lower care for the environment (19), which in our opinion, may be an obstacle to connecting with nature [considered a predictor of well-being, see (20)]. On the other hand, Catholicism is one of Christianity's main branches.

Although many people may subjectively believe that religion is important to their quality of life, it has not always been proven to have a beneficial associations with objective measures of well-being. Büssing, et al. (11) found that praying to God and relying on God's help were not significantly related to physical and mental indicators of health and quality of life. This refers to the utilization of one's religiosity as a resource to cope. In contrast, addressing religious struggles (a construct similar to negative religious coping), Koenig, et al. (20) and Lucchetti and Lucchetti (21) have shown that religious and spiritual struggles impair mental health and lower life satisfaction. In line with this, spiritual dryness, a specific form of spiritual crisis related to God, is negatively related to mental well-being and life satisfaction in Catholic pastoral workers in secular Germany (22, 23) and also in Catholic lay persons from religious Italy (24). This means that during spiritual struggles the resource religiosity is utilized and one can find significant associations with impaired mental well-being. Similarly, Bockrath, et al. (25), in their meta-analysis of longitudinal studies, showed that religious and spiritual struggles lead to worsening psychological adjustment. Finally, Konaszewski, et al. (26) observed that the instrumental use of religion can reduce quality of life and be associated with poorer physical functioning and lower well-being. Given these complexities, it seems necessary to undertake further work to determine the actual connection of religion and individual religiosity with well-being and life satisfaction.

As an integral part of the lives of many people around the world, religion is particularly prominent in the context of coping with stress and major life events. Religious coping, defined as “the degree to which religion is a part of the process of understanding and dealing with critical life events” (27) (p. 482), encompasses a range of cognitive and behavioral techniques that help an individual cope with or adapt to difficult life situations. In this sense, religion is more than a defense mechanism because it may provide people with a comprehensive and integrated framework of meaning to explain difficult life events in a way that is satisfactory to them. Findings by Pargament (28), the developer of the concept of religious coping, indicate that religious coping can be helpful or harmful, depending on the strategies used. In general, religious coping strategies are divided into two overarching broad categories: (1) positive religious coping and (2) negative religious coping (29). The former refers to a secure relationship with God, positive God concepts, and a sense of spiritual connection with a supportive religious community. It encompasses a repertoire of behaviors that are beneficial for people who experience stressful events (28). Negative religious coping, on the other hand, is characterized by an insecure relationship with God, negative God concepts, and an often offensively perceived religious community, and indicates the presence of interpersonal tensions, which, in light of past research, is generally maladaptive (30, 31). An example of positive religious coping is seeking religious and spiritual support, whereas negative coping involves evaluating an event as God's punishment or the work of the devil (32). Previous data indicate that positive religious coping is positively associated with well-being and life satisfaction (33–35). Conversely, negative religious coping is related to a diminished quality of life, poorer physical and social functioning, vitality, and mental health; and related to higher perceived stress, anxiety, and depression (32, 36, 37).

Religious coping is a social and cognitive resource that may influence well-being, life satisfaction, and perceived stress (29). However, the ongoing scientific discourse has overlooked the role of experiencing nature as a source to stand in wondering awe and to connect with the Sacred (38), which can enhance the effect of beneficial adaptation caused by positive religious coping. We should note that numerous experimental studies have shown strong evidence of links between experiencing nature and recovery from physiological stress and mental fatigue (39). Moreover, contact with nature has been shown to mediate the negative effects of stress, thus reducing negative mood states and, most important, enhancing positive emotions (40). Büssing, et al. (41) noted that spending more time outdoors and experiencing nature during the COVID-19 pandemic can help find meaning in life, reflect on what is important in life, and revaluate important aspects of one's life in order to be more aware of one's environment and other people and to deal more consciously (mindfully) with adversity. In another study, Büssing, et al. (42) found that the themes of recognizing meaning in life, having (religious) trust and having stable relationships, mindful encounters with nature, and time spent on reflection helped people cope with limitations imposed during the COVID-19 pandemic and allowed them to better enjoy silence and focus on their own spiritual resources (i.e., prayer or meditation). In addition, the effects of perceiving nature and enjoying times of silence on well-being have been shown to be mediated by perceptions of wondering awe and subsequent feelings of gratitude (38). On the other hand, in Rosmarin, et al.'s (43) study, religious coping (but only positive coping) was a key predictor of gratitude.

In a recent study, Büssing, et al. (41) found experiences of nature and time spent in silence (also as a part of spiritual experience and practice, e.g., praying and contemplation) favor the phenomenon of spiritual transformation. According to this theory, spiritual experiences are brief states of transcendent consciousness that are often accompanied by intense feelings of awe and bliss and a sense of oneness with the universe (44). Spiritual transformation itself represents a “fundamental change in the place of the sacred in life of the individual” that involves a “radical reorganization of identity, meaning and purpose in life” (45) (*p*. 21). For example, in Kremer and Ironson's (46) study with HIV infected people, spiritual transformation was associated with enhanced well-being, less stress, and better coping. In addition, Zarzycka and Zietek (8) found that spiritual transformation mediated the relationship between religious struggles and life satisfaction. Even spiritual struggles—when they are overcome—can change a person's perspective and behaviors (47). In other studies, positive religious coping fostered a sense of spirituality and transcendence, which consequently appeared to facilitate perceptions of a connection with nature and other people, as well as of times spent in silence (38, 48). In terms of spiritual transformation, these perceptions can have consequences for one's level of functioning and changed attitudes and behaviors (49). Self-transcendent emotions can also intensify a person's level of spirituality and religious feelings (50). Thus, it is highly likely that the search for a sense of spiritual connection (a part of positive religious coping) can express itself through a bond with nature. Thus, it seems that perceptions of nature and the ability to enjoy times of quietness (also in nature) can enhance the relationship between positive religious coping and quality of life.

The purpose of this study was thus to evaluate the relationships among religious coping, experiences with nature and times spent in silence, and well-being and life satisfaction. On the basis of the literature reviewed above, we hypothesize that positive religious coping will be positively, and negative religious coping will be negatively related to mental health i.e., well-being and life satisfaction; (Hypothesis 1). In addition, we hypothesize that more frequent experiences with nature and times spent in silence will mediate the association between positive religious coping and well-being and life satisfaction (Hypothesis 2).

## Materials and methods

### Participants and procedure

The study was conducted among Polish Catholics in 2021–2022 with the approval of the ethics committee of the Institute of Psychology of the Polish Academy of Sciences in Warsaw (No. 15/4/2021). Data from anonymous online questionnaires were collected on the Google Forms platform. Before completing the questionnaires, each participant gave informed consent. The invitation to participate was distributed *via* social media and in national media (newspapers and online portals). The two criteria for recruitment were (a) age > 18 years and (b) a self-reported Catholic religious affiliation. Incomplete data (i.e., 12 cases) were excluded from the analyses. The final sample consisted of 1,010 Catholic participants from Poland [ages *M* = 36.50, *SD* = 8.29 (18 to 73); 61% women].

The survey procedure consisted of completing questionnaires on religious coping, experiences of nature and times spent in silence, and well-being and life satisfaction. The average time to participate in the survey was 8 min.

### Measures

We used the Brief RCOPE (29), which has been standardized in Polish (51), to measure religious coping. The scale is used to evaluate one's strategies for coping with life stressors. It consists of 14 statements that load on two factors: (a) positive religious coping (α = 0.92; all Cronbach's alpha coefficients are for the current study data) and (b) negative religious coping (α = 0.81). Positive religious coping refers to a positive relationship with a transcendent force, a spiritual connection with others, and a benevolent attitude toward the world, whereas negative religious coping reflects spiritual tension and perceived difficulties in one's relationships with the self, others, and the transcendent force (29). The participants were asked to respond to each Brief RCOPE statement on a five-point Likert scale that ranged from 0 (*not at all*) to 4 (*a great deal*).

Experiences of nature and times spent in silence were assessed with the respective *Nature/Silence* subscale of the Perception of Change Questionnaire (PCQ) by Büssing, et al. (41). This subscale consists of four statements: (a) “I go outdoors much more often”, (b) “I consciously take more time for silence”, (c) “I perceive nature more intensely”, and (d) “I more enjoy quiet times of reflection”. Participants rated each statement (compared to last year) on a five-point Likert scale that ranged from 0 (*strongly disagree*) to 4 (*strongly agree*). The original version of the PCQ was translated into Polish by two independent translators. The translations were adjusted to the final version of the scale by the present authors. Next, the final version was back-translated into English by two independent translators with a high level of proficiency in English. Any differences between the original and back-translated versions of the scale were discussed and amended by three authors of the study, and the final version was deemed accepted by the scale's author. The translation of the scale was done according to accepted principles developed for the purposes of intercultural research. Exploratory analysis of the Polish language version of the PCQ subscale showed that it has a unidimensional structure, with this factor explaining 74.33% of the variance. The Polish version of the PCQ subscale *Nature/Silence* also showed good internal consistency (α = 0.89).

We used the World Health Organization's five-item Well-Being Index (52), in Polish (53), to measure mental well-being. This instrument is a unidimensional questionnaire that measures positive well-being, that is, positive mental health during the past 14 days. The single-factor scale (α = 0.89) consists of five statements in self-report form. The respondent indicates their attitude toward each statement on a six-point scale that ranges from 0 (*none of the time*) to 5 (*all of the time*). The scale has good psychometric properties (52). Sample statements include: “I have felt calm and relaxed” and “I woke up feeling fresh and rested”.

We used the Brief Multidimensional Life Satisfaction Scale (9), Polish (54) version, to measure multidimensional life satisfaction. This scale consists of 10 statements (α = 0.91). General satisfaction with life consists of five domains, for example, internal (me, all of life), external (work, where I live), professional (financial situation, future prospects), social (friendships, family life), and health related (health situation, ability to deal with everyday life). The respondent rates their satisfaction on a seven-point Likert scale that ranges from 1 (*very dissatisfied*) to 7 (*very satisfied*).

In addition, participants were asked to identify their religiosity and spirituality on two separate Likert scales that ranged from 1 (*I am not at all religious/spiritual*) to 5 (*I am very religious/spiritual*).

### Statistical data analysis

Statistical data analysis was conducted using IBM SPSS Statistics (Version 27) and IBM SPSS Statistics Amos (Version 27). We used a Kolmogorov–Smirnov (the results are shown in Table 1) to verify the normality of distribution and Levene's test to assess the homogeneity of variance. The results allowed us to apply parametric tests. We used Pearson's correlation analysis and structural equation modeling (using the maximum likelihood estimation) to determine the relationships between the variables. To assess the model's fit to the data, the following indices were used: goodness-of-fit index (GFI), comparative fit index (CFI), root-mean-square error of approximation (RMSEA), and relative chi-square (χ^2^/*df*). GFI values ≥90 and CFI values ≥95 indicate good adjustment of the model to the data; χ^2^/df values <2 also suggest a good fit of the model to the data. RMSEA values <0.08 can also be interpreted as a good fit to the data (55). To verify the mediating role of nature and silence on the relationship between religious coping and well-being and life satisfaction, we conducted a bootstrapping analysis to establish 95% confidence intervals (CIs) for the estimated effects.

**Table 1:** Means and correlations.

**Variable**	***M* (*SD*)**	**KS**	**1**.	**2**.	**3**.	**4**.	**5**.
1. Positive religious coping	15.11 (6.49)	1.48	—				
2. Negative religious coping	11.11 (3.86)	1.02	0.36***	—			
3. Perception of nature and silence	6.84 (3.61)	1.13	0.25***	−0.03	—		
4. Life satisfaction	37.27 (12.85)	1.07	0.18***	−0.14***	0.19***	—	
5. Well-being	13.77 (5.41)	1.53	0.10**	−0.07*	0.17***	0.33***	—
6. Spirituality	3.41 (1.18)	0.99	0.59***	0.16***	0.19***	0.15***	0.12***
7. Religiosity	3.23 (1.33)	1.03	0.61***	0.20***	0.18***	0.13***	0.11***

N = 1,010, KS = Kolmogorov-Smirnov Test for Normality (each time insignificant statistics).

^*^p < 0.05, ^**^p < 0.01, ^***^p < 0.001.

## Results

Results regarding life satisfaction, well-being, and perception of nature and silence indicated moderate average levels of these phenomena among the participants (Table 1). The mean positive religious coping score fit within the limits of sten five and can be defined as average coping, and the mean negative religious coping score is within the third sten and can be described as a low tendency toward taking advantage of this means of coping.

A correlation analysis revealed statistically significant relationships: positive religious coping was strongly positively related to spirituality and religiosity, weakly positively correlated to negative religious coping and perception of nature and silence, and marginally positively to life satisfaction and well-being. In contrast, negative religious coping was marginally negatively associated with life satisfaction, well-being, and positively weak related to spirituality and religiosity. Perception of nature and silence was positively marginally related to spirituality and religiosity. The means, standard deviations, and correlation coefficients are presented in Table 1. Moreover, age was marginally associated with higher rates of positive religious coping (*r* = 0.15, *p* < 0.001). Gender (0 = male, 1 = female) did not correlate at a statistically significant level with the results.

**Figure 1:**
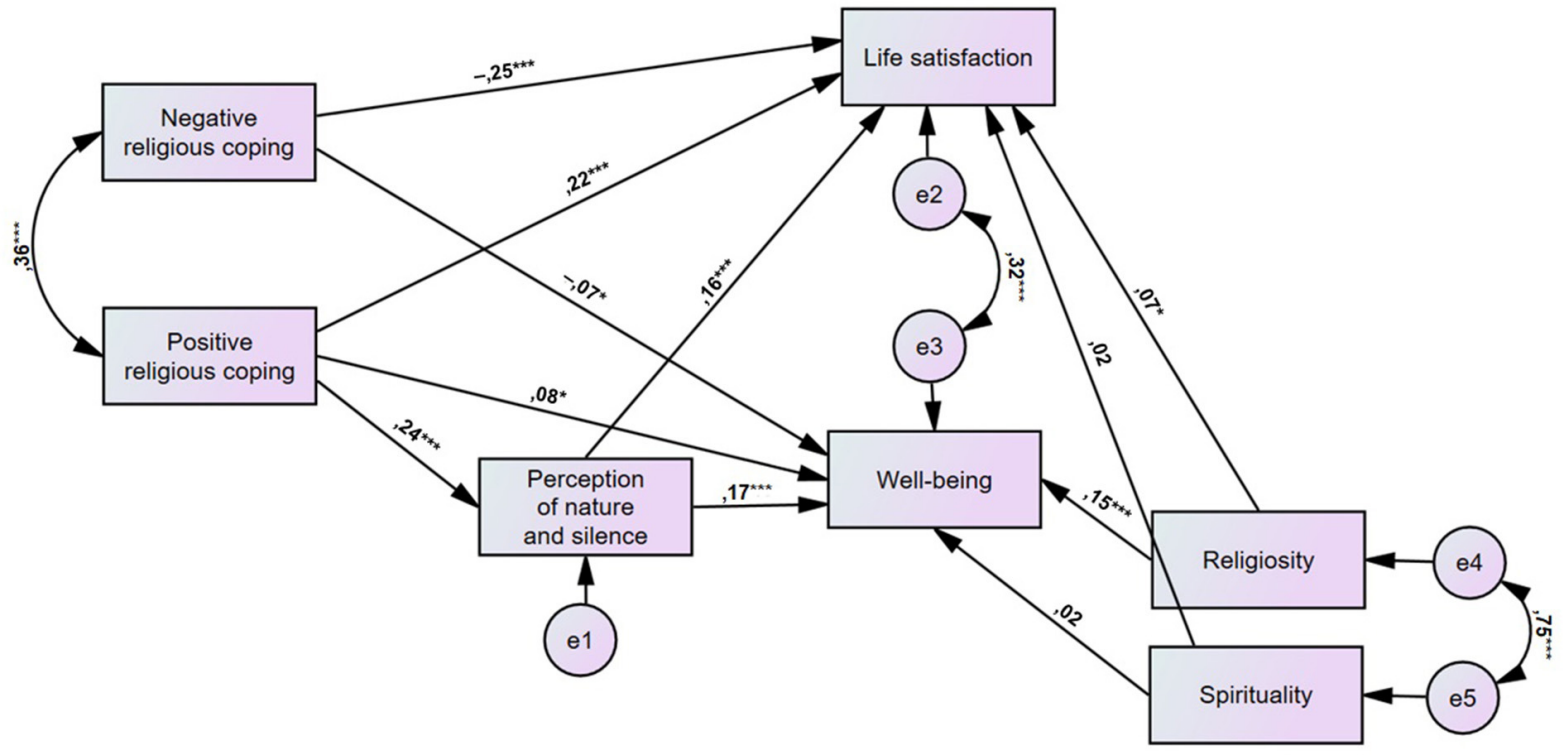
Study model: Relationships between religious coping, perception of nature and silence, satisfaction with life, and well-being (*N* = 1,010; *p < 0.05, ***p < 0.001).

We then used structural equation modeling to examine the hypotheses. In addition, we included religiosity and spirituality as covariates in the model (but did not include age and gender due to the lack of statistically significant associations of these variables with well-being and life satisfaction in the correlation analysis described earlier). The model was found to be well–suited to the data: χ^2^(7) = 7.48, *p* = 0.381; χ^2^/*df* = 1.07, RMSEA = 0.001, 90% CI [0.000, 0.055], GFI = 1.00, adjusted GFI = 1.00, CFI = 1.00. Figure 1 presents the standardized path coefficients: for one-direction arrows, these are standardized regression coefficients, and for two-direction arrows these are correlation coefficients. Combined positive and negative religious coping, as well as perception of nature and silence (while controlling religiosity and spirituality) explained 19% of the variance concerning life satisfaction and 14% of the variance concerning well-being.

There was a positive direct relationship of positive religious coping with life satisfaction (β = 0.22, *p* < 0.001) and well-being (β = 0.08, *p* = 0.011). The direct relationship of negative religious coping with life satisfaction was negative (β = - 0.25, *p* < 0.001) and this was also the case for well-being (β = −0.07, *p* = 0.040). A direct relationship between positive religious coping and perception of nature and silence was also observed, and the relationship was positive (β = 0.24, *p* < 0.001). Furthermore, the direct relationship of perception of nature and silence on life satisfaction was positive (β = 0.16, *p* < 0.001), and this was also the case for well-being (β = 0.17, *p* < 0.001). Finally, the results of the mediation analyses indicated that perception of nature and silence played a mediating role in the relationship between positive religious coping and both life satisfaction and well-being. The indirect relationship of positive religious coping with life satisfaction through perception of nature and silence was partial and positive (β = 0.04, *p* = 0.008), and this was similar for well-being (β = 0.04, *p* = 0.011). The value of the regression coefficients of the covariates (spirituality and religiosity) on the outcome variables is shown in Figure 1. For comparison, we examined a model in which perception of nature and silence could mediate the relationship between negative religious coping and life satisfaction and well-being. However, the model fit was not acceptable: χ^2^(7) = 148.23, *p* < 0.001; χ^2^/*df* = 21.18, RMSEA = 0.217, 90% CI [0.167, 0.271], GFI = 0.881, adjusted GFI = 0.712, CFI = 0.857.

## Discussion

The purpose of this study was to assess the relationships among religious coping, perceptions of nature and silence, and life satisfaction and well-being. As we hypothesized, positive religious coping (i.e., working together with God as partners and looking to God for strength, support, and guidance) was positively associated quality of life to some extent, and negative religious coping (i.e., wondering whether God has abandoned you, expressing anger at God), works in the opposite direction as it was associated with reduced well-being and life satisfaction [Hypothesis 1], which corresponds to the literature enrolling religious people (32–37).

The obtained results suggest that religious coping may encompass a framework for assigning meanings related to the reduction of psychological suffering and the pursuit of value-based mental well-being. The data obtained, however, could also be explained by other functions of religious coping: comfort, control, identity, spiritual connection, and intimacy with others (29, 51). It is interesting to note that previous research points to the critical role of religious coping in adaptation not only in the general population but also in clinical groups. For example, in a study conducted in the United States by Koenig (56), almost half of hospitalized patients spontaneously reported that ozzne or more religious factors helped them accept their illness and develop a sense of subjective well-being, and the vast majority of participants admitted that they used religion as a coping strategy. Also in secular Germany, patients with chronic diseases used their religiosity/spirituality to cope with illness and suffering (5, 11). Hebert, et al. (36), on the other hand, noted that negative religious coping was far less prevalent among women with breast cancer and that it had an impact on worsening overall mental health, depressive symptoms, and lower life satisfaction. In our study, however, we did not assess the level of intensity of religious identity or its centrality in life. According to Aten, et al. (57), this variable may increase the association between positive religious coping and well-being given that any turn toward faith entails a stronger sense of belonging to a group.

The relationship between experiencing nature and health is a common observation in the literature (40), but little is still known about the mechanisms at work in this regard (58). In our study, we confirmed the hypothesis that the health benefits of experiencing nature and spending and enjoying time in silence may be driven by positive religious coping (Hypothesis 2). In other words, people who favor religious coping strategies such as surrendering to God's will, seeking support from one's church, being faithful to religious practices, and positive religious re-evaluation may be more open to experiencing contact with nature as a place to encounter the Sacred in their life, and may be more likely to spend time in silence reflecting (as it is practiced for example in religious contemplation and prayer times), which may consequently promotes their life satisfaction and well-being. This perception of nature may be comparable to the phenomenon of spiritual transformation, which is considered an effect of adaptive forms of coping and is the result of positive transformations of life assumptions (44, 45). However, such a transformation requires stronger stimuli: Walking in the forest alone may not be enough, it requires deep moments of wondering awe which can of course be triggered by experiences in nature (38). In another study, Büssing, et al. (59) noted that people who said they relied on their faith as a strong foundation manifested more frequent feelings of awe and gratitude, as well as stronger perceptions of nature and more time spent in silence, which also supports our findings. In addition, Wnuk and Marcinkowski (60) showed that positive religious coping promotes positive affect. On the other hand, Mayer, et al. (61) found that positive affect was significantly correlated with a sense of connection to nature, and they explained why contact with nature can lead to increased reflection. The identification of experiencing nature with spiritual transformation has also been developed on theological and philosophical grounds. John Paul II spoke of connecting with God through nature: “Indeed, the feeling of joy that fills us when we admire this marvelous landscape directs our thought toward God, who, looking at the first creation, rejoiced in the work of His hands” (62). This underlines from a theological point of view that the Sacred can be encountered in nature, too—given one is able and willing to perceive it. It seems that religiously active people are more aware of this resource (38, 59). The book of Genesis says so. “God saw that they were good” (cf. Genesis 1:10). Can we not feel surrounded here by the love of God, who opens the book of nature to us, inviting us to read in it the signs of His presence and goodness (62)?

In addition, our findings indirectly correspond with those of Myers (63), according to whom mindfulness (a construct related to conscious awareness of nature and enjoying times of silence and reflection) mediates the relationship between religious coping and emotion regulation, as well as Salmani and Zoqi's (64) findings, which indicate that mindfulness explained the relationship between spiritual intelligence and well-being. In summary, the data obtained indicate that perceptions of nature and time spent in silence that promote reflection can mediate the positive impact of positive religious coping on a person's quality of life.

Our analyses indicate that the level of positive religious coping are higher in older people. This might be explained by differences in religious socialization which can be observed also in Catholic Poland, instead of religious development processes. We should note that correlational studies have consistently shown a relationship between age and the use of religious beliefs or activities for coping with stress (65, 66). Yet, it remains to be shown that the current, usually less religious, generations will become more religious in their later life. Although the exact mechanism has yet to be demonstrated, religious beliefs associated with positive religious coping may influence the way one copes with illness and suffering. At least religions provide a framework for how illness and suffering can be interpreted (67). Alternatively, religious beliefs may shape self-esteem in a more resilient way than other sources that decline with increasing age and declining health (68).

In addition, we observed associations of religiosity and spirituality with religious coping, well-being, and life satisfaction, which is a common observation in the literature (29, 69). We have also shown that both spirituality and religiosity are positively associated, to some extent, with perceptions of nature and silence. In contrast, the perception of wondering awe is much higher in people with a spiritual/religious background and thus more frequent practices that may sensitize these individuals for these perceptions (38, 59). According to Skalski, et al. (70), spirituality reflects a personal relationship with the divine and thus positively predicts a sense of connection with nature. On the other hand, the literature on the relationship between religion and nature has been inconclusive (71). Recent findings, however, indicate that religion can foster perceptions of nature, and any negative relationship between these variables is fully explained by religious fundamentalism and support for authoritarianism (70). We should also note that Pope Francis, as the leader of the Catholic Church, has pointed out in one of his encyclicals that it is necessary to combine concern for the environment with a sincere love of humanity and with an ongoing commitment to society's problems (72). Contact and communion with nature helps to seek and experience God. In a speech in Denver to young people (August 14, 1993), the Pole John Paul II stated: “By reading the book of nature, reason can come to a knowledge of God—a personal God, infinitely good, wise, powerful and eternal, who is transcendent to the world, but at the same time present in the heart of his creatures… This is how contemplation of nature reveals not only the Creator, but also man's role in the world He created” (62).

## Limitations

Despite its strengths, however, this study has some limitations. First, the data came only from Catholics in Poland [according to common estimates, about 80% of Polish citizens are Catholic; see (69)] and can thus not easily be transferred to other religious groups. Other religions (e.g., Islam, Hinduism, Buddhism, Shintoism) may offer their followers different conceptions of nature and the relationship between humans and the environment (73). Second, the data were collected during the COVID-19 pandemic which might have affected respondents' mental health. Although there were no specific restrictions in place when the study was conducted in Poland, we assume that exposure to information about COVID-19 (e.g., media coverage of the number of hospitalizations or deaths), as well as the recent lockdown experience, could have influenced the averaged levels and/or associations of the variables. Third, the study is correlational in nature and thus making direct judgments about the causes and effects of phenomena is not appropriate. In future research, the use of experimental studies with manipulation of nature exposure seems particularly worthwhile. Further research is also needed to determine the actual relationship of perception of nature and spiritual transformation, as well as potential mediators and facilitators for this relationship.

## Conclusion

Our study provides new data on the role of perceptions of nature as a place of encounter and enjoying times silence in the link between religious coping and well-being and life satisfaction as outcomes. We are among the first to show that perceptions of nature and times of silence underlie the relationship between religious coping and well-being and life satisfaction outcomes. The data indicate that positive religious coping is an important resource that may contribute to well-being and life satisfaction among Catholics, both as an independent resource (main effect) and indirectly through its relationship with perceptions of nature and silence.

There is growing interest in psychology and pedagogy in regard to integrating religious beliefs and practices with therapy (74, 75). For example, a meta-analysis conducted by Kaplar, et al. (76) suggested that interventions involving religious resources are effective in improving mental health. How future counseling interventions could contribute to promoting positive religious coping strategies and train awareness of nature as a resource—that could finally lead to a spiritual transformation—remains to be clarified. Such efforts would be consistent with the literature for example on forgiveness interventions that promote the release of negative and promotion of positive emotions (77) or of mindfulness training on mental health conditions (78). Nevertheless, even if affected people struggle with God or feel abandoned by God (which could be attributed to negative religious coping), they can overcome such phases of spiritual dryness when they are adequately supported (47). In terms of spiritual transformation, after overcoming these phases they may later experience “deeper spiritual clarity and depth” and be ready “all the more to help others” (47).

## Data availability statement

The original contributions presented in the study are included in the article/supplementary material, further inquiries can be directed to the corresponding author.

## Ethics statement

The studies involving human participants were reviewed and approved by the Ethics Committee of the Institute of Psychology of the Polish Academy of Sciences. The patients/participants provided their written informed consent to participate in this study.

## Author contributions

Methodology: SS-B, KK, and JS. Formal analysis: SS-B, KK, and AB. Investigation: SS-B and LT. Writing—original draft preparation: SS-B. Writing—review and editing: SS-B, KK, LT, AB, and JS. Project administration and funding acquisition: AB and JS. All authors have read and agreed to the published version of the manuscript.

## Conflict of interest

The authors declare that the research was conducted in the absence of any commercial or financial relationships that could be construed as a potential conflict of interest.

## Publisher's note

All claims expressed in this article are solely those of the authors and do not necessarily represent those of their affiliated organizations, or those of the publisher, the editors and the reviewers. Any product that may be evaluated in this article, or claim that may be made by its manufacturer, is not guaranteed or endorsed by the publisher.
